# Sickness Prevented

**Published:** 1875-11

**Authors:** 


### SICKNESS PREVENTED.

It may seem a singular fact, but it *is* a fact, nevertheless, that intermittent fevers, remittent fevers, agues, and all other diseases which take the form of "malarial" disorders, are more general in a very dry or a very damp season, than in any other. This shows that it is not only in slaking our thirst that water concerns us. Ever under our feet ebbs and flows the subsoil tide, freighted with good or evil, as we use it well or ill. The earth, even under natural conditions, becomes loaded with vegetable *debris*, and to these, in settled districts, is added the soakage of animal refuse and garbage of various kinds. For the decomposition of organic matter, two things are requisite — heat and moisture; the first of which is afforded by the sun, the second, if we permit it, by this subsoil water. In an undrained, saturated soil, we have alternately an elevation of the level of our underground sea during a wet season, and a gradual lowering by evaporation. In the former case a previously dry layer of organic matter being moistened for the sun's fermenting influence; in the latter, a lower layer, which did not decompose while it was wholly submerged, being left uncovered in just a sufficient state of dampness to favor putrescence. In either case malarial emanations arise, poisoning the atmosphere and undermining health.

The agues which are very prevalent in some localities are directly due to this soil saturation; and, besides such acute manifestations of its action, there are scores of cases of neuralgia, dysentery, and obscure chronic maladies, which every educated physician knows to be caused by persistent malarial blood-poisoning, all of which might be easily prevented by proper drainage. Free access of air oxidizes rapidly, and renders harmless decomposing substances, and in an ordinarily porous soil there should be a constant circulation of air, so that in draining we also ventilate the earth.

If to the vegetable decomposition just considered we add animal matter, more especially if we receive and concentrate the miasmatic vapors in our houses, we run graver risks than that of "chills." Typhoid fever, diphtheria, cerebro-spinal meningitis, and other dangerous disorders, are too often associated with fermentation in damp cellars,— the former two depending, apparently, upon excremental pollution. Even where specific disease is not thus directly caused, the indoor exhalations from undrained cellars lower the vitality of the inmates, so as to increase their susceptibility to any passing contagion. It is curious to note how, in the city of New York, the march of nearly every epidemic follows the lines of the obstructed water-courses and undrained swamps over which many houses are built; and in less populous places doctors have learned to look for greater frequency and severity of contagious diseases under similar conditions.

The farmer who undertakes to raise vegetables soon learns the importance of draining his soil; but he who has a family to raise seems to regard human growth as requiring no particular cultivation. While this matter of drainage is the first great step for all communities to take, it is none the less necessary to individuals—the residents of the detached villa and farmhouse. Wherever and whenever an excavation is made in which to construct a cellar for a house, there necessarily occurs an interruption of the natural drainage of the soil. The underground channels for the percolation of water are intercepted, and must be restored by the construction of a drain below the level of the cellar, and all the surrounding area requires a system of drains connecting with the main outlet.

Another fruitful source of soil saturation and malarial poisoning of whole neighborhoods, is the common method of damming streams for manufacturing or other purposes, whereby the water is raised far above its natural bed, and soaks into the soil for a wide distance on every side. This could be avoided by confining these mill-ponds to a properly defined space, from which all vegetable matter should be carefully removed, and the sides protected by walls from contact with vegetation.

---

### POMARIUS.

This article, introduced by the Health Food Company, No. 137 Eighth Street, is pronounced delicious by all who use it. It is certainly a very palatable and wholesome sub-acid fruit product, agreeing perfectly with the weakest stomachs. It is put up in glass, at 50 cents and $1.75,—the large jar holding precisely five pounds. It is cheaper than fine currant-jelly, and is, to our mind, more desirable.

---

Idlers can not even find time to be idle, or the industrious to be at leisure. We must be always doing or suffering.

